# Assessing the impact of shallow subsurface pipe drainage on soil salinity and crop yield in arid zone

**DOI:** 10.7717/peerj.12622

**Published:** 2021-12-14

**Authors:** Haichang Yang, Weiye Chen, Yun Chen, Fenghua Zhang, Xiaohu Yang

**Affiliations:** 1 Agricultural college, Shihezi University, Shihezi, Xinjiang, P. R. China; 2 Land and Water, CSIRO, Canberra, ACT, Australia

**Keywords:** Soil salinization, Drip irrigation, Plastic mulch, Growth stage, Desalinization

## Abstract

**Purpose:**

Soil salinization is one of the key problems of sustainable development of arid agricultural land. Exploring the use of shallow subsurface pipe drainage to improve soil salinization.

**Methods:**

This study investigates the desalinization effect of shallow subsurface pipe drainage, in combination with drip irrigation under plastic mulch, in an arid region in China. Field data collection was conducted in 2010. Soil salinity at a range of soil depths, water EC and pH of subsurface pipe drainage and crop yield during crop growth stages in salinized farmlands were measured.

**Results and Conclusion:**

The results show that soil salinity was reduced significantly on mildly (1–3 dS m^−1^) and moderately (3–6 dS m^−1^) salinized farmlands. The highest desalinization rate of mildly and moderately salinized soils was 51% and 91% respectively. The desalinization in upper soil layers, to a depth of 60 cm, was more significant than that in lower soil layers. Drainage water salinity was much higher than irrigation water salinity. Crop yield on mildly and moderately salinized land increased about 25% and 50%, respectively. This indicates that the combination of drip irrigation and shallow subsurface pipe drainage on farmlands is potential feasible to desalt farmlands and to improve crop yield. The study has led to a desalinization of 330 ha year^−1^ in Xinjiang.

## Introduction

Secondary soil salinization is caused by anthropogenic factors which raise the water table and move salt into the root zone (Aslam et al., 2006; Carter et al., 2012). It becomes more significant due to water resources scarcity, ecological vulnerability and unreasonable resource utilization. Many investigations have been carried out to tackle this global problem. These approaches include the control and rehabilitation of salinized lands by engineering measures (Aidarov & Pankova, 2007) and ecological restoration (Tzanopoulos, Mitchley & Pantis, 2007) in arid desert regions, monitoring soil salt and water content changes and water-salt balance under different improvement measures (Pannell & Ewing, 2006; Xie, Liu & Sun, 2011), as well as the evaluation and simulation of soil salinization using remote sensing technology (Metternicht & Zinck, 2003) and mathematical models (Tweed et al., 2007). A range of measures can be employed to alleviate salt stress to varying extents. The most commonly used agricultural measures include soil replacement improvement (Moffat & Bending, 2000), subsoiling (Rengasamy, 2006), straw mulching (Pang et al., 2010), straw burying (Zhao et al., 2014) and paddy-upland rotation (Sugimori et al., 2008). Chemical amendments (Jones, Hauser & Popham, 1994; Liu, Yang & Yao, 2006) and biological control measures (Busch & Smith, 1993; Rai, 1991) are also efficient to improve salinized wastelands. However, many of these methods can only treat the symptom but not the cause. One of the main causes of a water-salt unbalance in arid regions is due to the water table rise at local and sub-catchment level due to inappropriate irrigation and drainage systems (Khan et al., 2007). There have been few systematic studies on integrating irrigation, drainage, soil salinity and crop production to evaluate the improvement of salinized farmland (Singh et al., 2006; Wang et al., 2008).

Subsurface pipe drainage removes excess groundwater. It aims to increase the rate at which water drains laterally and vertically from the soil, and provide favorable growing conditions in the root zone. By lowering the water table, the depth of dry soil is increased (Christen & Ayars, 2001). At the same time, soil salt is translocated in the irrigation water and is discharged through the subsurface pipe (Bouwer, 2000). Therefore, soil salinity is reduced. By controlling the groundwater table, soil properties, such as structure, porosity and soil moisture, are improved. Moreover, subsurface pipe drainage usually has a good leaching effect on soluble ions (Ai & Li, 2007). The longer the service life of subsurface pipes, the more obvious the increase of the aeration pores of soil, and the better the effect of drainage improvement (Skaggs, Breve & Gilliam, 1994).

The beneficial effect of subsurface pipe drainage has a direct impact on crop yield. Wang et al. (2006) analyzed the crop relative yield for different drain spacings. The impacts of excess water stress and planting delay on yield reduction increased with the wider drain spacing. Ma et al. (2007) simulated the effects of crop rotation, tillage and controlled drainage on crop yield using the Root Zone Water Quality Model (RZWQM). However, studies of the effect of subsurface pipe drainage on crop yield have a limited coverage in the scientific literature.

Since 1996, drip irrigation under plastic mulch have been widely employed in Xinjiang, China. The original surface drainage gravity system was abandoned, due to no soil leakage. The drainage system for flood irrigation was replaced by drip irrigation without drainage, and a new water-salt balance was established. The initial improvement in soil salinization is now facing new challenges due to variable irrigation conditions and needs new methods and techniques. Most research has focused on engineering aspects of subsurface pipe drainage, such as drain depth and spacing. Few studies have assessed soil desalinization effects by taking drainage water quality into consideration. Much less is known about the desalinization effect of linking the shallow subsurface pipe drainage to drip irrigation under plastic mulch in the unique arid oasis environment of Xinjiang. Therefore, we present a field-scale case study which evaluates the impact of shallow subsurface pipe drainage on soil salinity and crop yield in Xinjiang with following specific objectives: (1) to analyze the variations of drainage water EC and pH, (2) to quantify the effectiveness of desalinization at different soil depths and growth periods, and (3) to compare the improvement of crop yield on mildly and moderately salinized farmlands.

**Figure 1:**
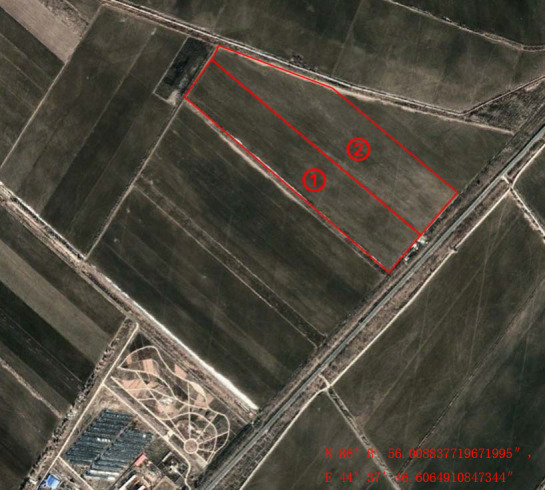
Landmasses of study area. (1) Mildly salinized farmland (1–3 dS m^−1^), (2) Moderately salinized farmlands (3–6 dS m^−1^).

## Materials & Methods

### Study area

The study area is situated at Shihutan Township (85°52″–86°12″E, 44°31″–44°46″N, Fig. 1), Shihezi Oasis, Xinjiang Uyghur Autonomous Region, China. Covering an area of 20 ha, the experimental site is located in south margin of the Gurbantunggut Desert where severely salinized farmlands have been abandoned for 14 years. The annual average rainfall is 175 mm (1956–2010). The annual average temperature is 6.8 °C. The annual average evapotranspiration (ET) is 1200 mm (1956–2010). The groundwater table varies between 1.5 m to 3.5 m during the year in response to irrigation and evapotranspiration. The mineralization degree of groundwater is 5-7 g L^−1^. The average mass fraction of soil organic matter is 11.4 g kg^−1^, and the mean of soil pH value is 8.28. Secondary soil salinity decreases from east to west, with the highest reaching over 5 g kg^−1^ and the lowest about 1.5 g kg^−1^. Soil physical parameters at different depths are shown in Table 1. The crop type was cotton. The groundwater level at the midpoint of the distance between drainage pipes is 1.5 ∼2 m high in spring, and 2.5 ∼3.5 m low in summer and autumn.

**Table 1:** Soil physical parameters.

Soil layer (cm)	Soil texture^*^	Volume moisture content (%)^**^	Bulk density (g cm^−3^)	Porosity (%)
0–25	Medium loam	15.33	1.205	54.53
25–50	Medium loam	16.91	1.559	41.19
50–70	Heavy loam	21.05	1.601	39.59
70–90	Heavy loam	20.26	1.582	40.31
90–110	Sandy loam	22.82	1.716	35.27
110–130	Medium loam	26.19	1.536	42.04

**Notes.**

* Soil texture using Katschinshi system.

** Volume moisture content means absolute soil moisture.

### Field experiment

#### Implementation of subsurface drainage systems

**Figure 2:**
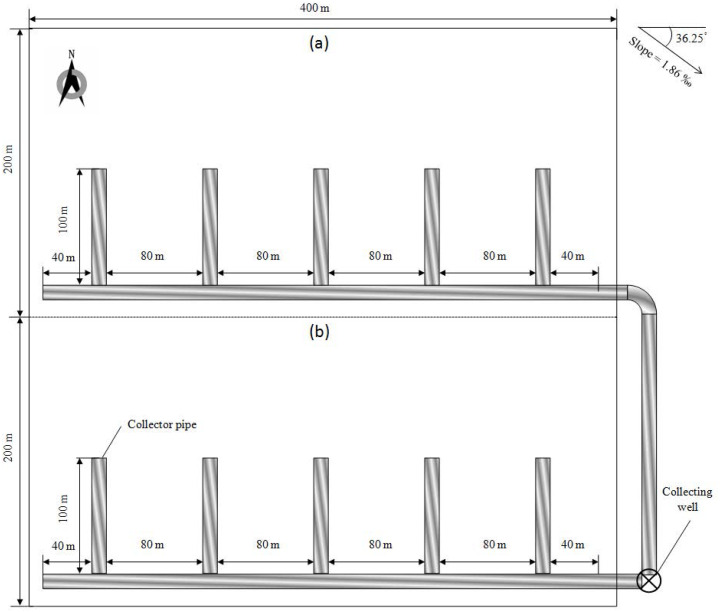
Schematic diagram of subsurface pipeline laying. (A) Moderately salinized soil. (B) Mildly salinized soil. Drain depth is 2 m.

Subsurface pipes were laid on the salinized farmlands in Autumn of 2009. The drainage in soils was digged using the trencher which had a great power source and special cutting knives or bits. A drain depth in agricultural practice is usually recommended as >1.2 m to avoid damaging the laterals (Tiwari & Goel, 2015). In this study, drain depth was set at 2 m underneath the frozen soil layer in Shihezi. Drain spacing was taken as 80 m. A schematic diagram of field experiment set-up is shown in Fig. 2. Corrugated pipes made of polyvinyl chloride (PVC) have an inner diameter of 11 cm. They as drain pipes were coated by gauze and then backfill and compaction. Numerous soil and gravel in different sizes were used for backfilling. Size of soil and gravel gradually decreases from bottom to top. The drainage water in the collecting well was pumped to the shelter forest.

#### Surface experimental design

**Figure 3:**
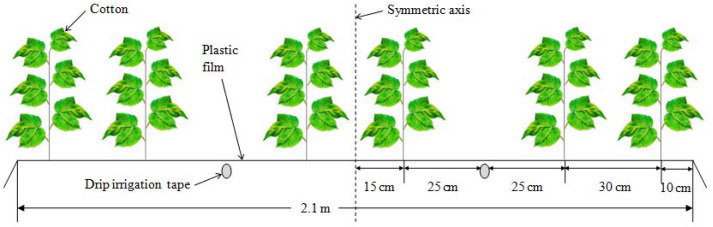
Schematic diagram of cotton planting pattern.

**Figure 4:**
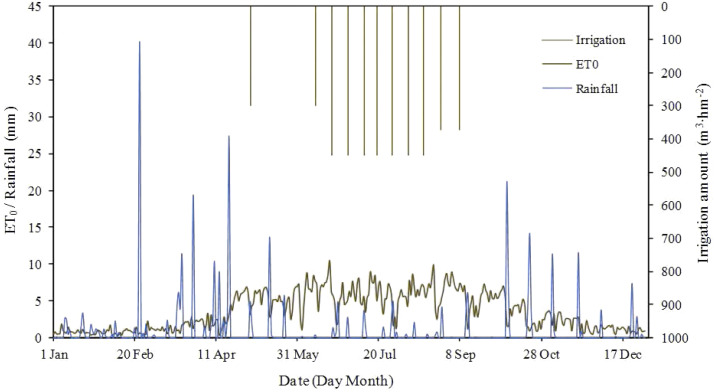
Rainfall, potential evapotranspiration and irrigation amount of experimental area in whole 2010.

The experimental cotton field laid with subsurface pipes was divided into a mildly salinized plot with a salt content of 1–3 dS m^−1^ and a moderate salinized plot with a salt content of 3-6 dS m^−1^. Each covers an area of 8 ha (400 m in length, 200 m in width). The mildly and moderately salinized soils without drainage pipes were used as two control check (CK) plots. Barrier was installed to prevent water movement between the experimental plots and the CK plots. One plastic film, two drip irrigation tapes and six rows of cotton were arranged for drip irrigation under mulch (Fig. 3). The film width was 2.1 m, and the cotton crop was irrigated 11 times during the whole growth stages. The total irrigation quota was 4.5 million L ha^−1^. Electrical conductivity (EC) of irrigation water was 0.35 dS m^−1^, and was assumed to have no impact on soil salinity. Rainfall and potential evapotranspiration (ET_0_) in the whole year are shown in Fig. 4. In this study, the total amount of nitrogen, phosphorus and potassium applied per acre of land during the growth of cotton is: nitrogen: 16.75 kg; phosphorus pentoxide: 2.76 kg; potassium oxide: 14.52 kg.

### Sample collection, measurement and analysis

(1) Sample collection: soil samples were collected from depths of 0–20 cm, 20–40 cm, 40–60 cm and 60–80 cm at the four primary growth stages, seeding, budding, flowering and bolling. There were three replications for mild salinization soil, moderate salinization soil and the CK plots. Drainage water in subsurface pipes was collected from a collection well using a manual water sampler. Soil electrical conductivity (EC_s_), electrical conductivity (EC_w_) and the pH value of drainage water were measured. Diagonal method was employed to select three sampling points to collect plant samples before the harvest of cotton on August 20. Table 2 shows the number of plants, number of bolls and mass of bolls were measured for calculating the cotton yield (kg ha^−1^). (2) Measurement: the EC of soil and drainage water in the subsurface pipes were measured by using an electrical conductivity meter (DDSJ-308A conductivity meter, water-to-soil ratio 5:1, dS m-1). The pH value of the drainage water was measured by Ampholine (LKB Producter AB, Sweden) pH meter (3310, water-to-soil ratio 2.5:1). Soil desalinization rate was calculated according to Eq. (1):

(1) \begin{eqnarray*}{D}_{r}= \frac{S{S}_{i}-S{S}_{f}}{S{S}_{i}} \times 100\text{%}\end{eqnarray*}

**Table 2:** Field investigation of crop yield components factors.

	Number of plants (plant ha^−1^)	Number of bolls (bolls plant^−1^)	Mean mass of bolls (g boll^−1^)
Mild salinization soil	1.56 × 10^5^	5.2	6.22
Mild CK	1.34 × 10^5^	4.9	6.14
Moderate salinization soil	1.57 × 10^5^	5.3	6.34
Moderate CK	1.11 × 10^5^	5.0	6.15

where *D*_*r*_ is desalinization rate of soil, *SS*_*i*_ is initial soil salinity, *SS*_*f*_ is final soil salinity.

Variation rate of drainage water salinity was calculated according to Eq. (2):

(2) \begin{eqnarray*}V{R}_{dw}= \frac{E{C}_{dw}-E{C}_{idw}}{E{C}_{idw}} \times 100\text{%}\end{eqnarray*}

where *VR*_*dw*_ is variation rate of drainage water salinity, *EC*_*dw*_ is EC of drainage water, *EC*_*idw*_ is initial EC of drainage water.

Cotton yield was calculated according to Eq. (3):

(3) \begin{eqnarray*}CY= \frac{{N}_{p}\times {N}_{b}\times {W}_{sb}}{1000} \end{eqnarray*}

where *CY* is seed cotton yield (kg ha^−1^), *N*_*p*_ is number of plants (plant ha^−1^), *N*_*b*_ is number of bolls (bolls plant^−1^), *W*_*sb*_ is mean weight of a single boll which is harvested randomly from different plants in rows of three sampling points (g boll^−1^).

### Statistical analysis

Statistical analysis was conducted using the statistical software SPSS version 20.0 (IBM, Armonk, NY). Data were reported as the mean ± standard error. Least significance difference (l.s.d.) was adopted for a variance analysis (Gong et al., 2015). The letter-marking method was used in the comparison of different treatments. Significant differences in ECs among different soil depths were indicated using different lowercase letters (*P* < 0.05). Significant differences in cotton yield between the treatments of subsurface pipe drainage and CK were indicated using different capital letters (*P* < 0.01) (Yang, Chen & Zhang, 2019).

## Results and Discussion

### Variation of soil salinity

**Figure 5:**
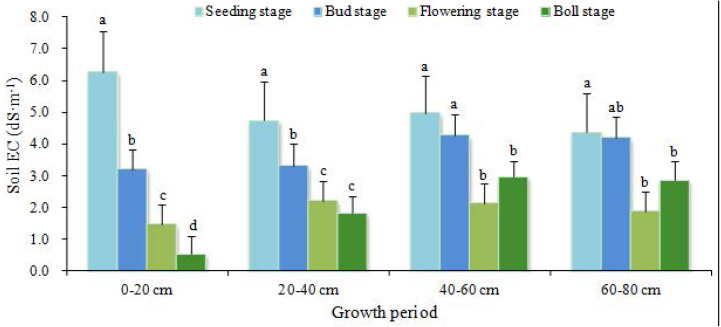
Salinity changes in the moderately salinized cotton field. Data show the mean ± standard error (*n* = 3). Significant differences (*P* < 0.05) among treatments are indicated by different lowercase letters.

Salinity of the moderately salinized soil decreased during all growth stages (Fig. 5). The decrease of ECs at the 0–20 cm depth was greater than those at other depths. Typically, the salt leaching effect was achieved through drip irrigation onto surface soil. After several applications of drip irrigation, soil salts migrated downwards with the wetting front, and the infiltration rate of surface soil was greater than those of the lower layers. ECs at the depths of 20–40 cm, 40–60 cm and 60–80 cm first decreased with time (before flowering stage) and then stabilized. From seeding stage to boll stage, the desalinization rates of the four soil layers were 91%, 61%, 40% and 34% from top to bottom of the profile, respectively. With an increase of irrigation times, the salt from the surface soil was leached downwards. Thus, subsurface pipe drainage was conducive to reducing soil salt and mitigating the adverse effect of soil salt on crop growth.

**Figure 6:**
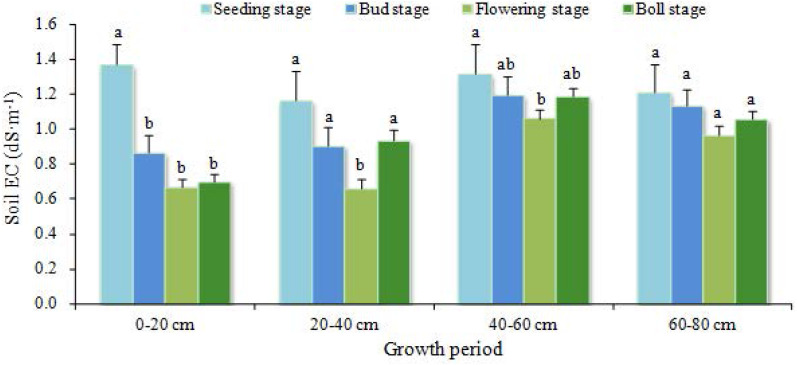
Salinity changes in the mildly salinized cotton field. Data show the mean ± standard error (*n* = 3). Significant differences (*P* < 0.05) among treatments are indicated by different lowercase letters.

ECs in mildly salinized cotton field shows a different pattern of variation during growth stages and at different soil depths (Fig. 6). At the 0–20 cm depth, ECs during seeding stage was much higher than at other stages (*P* < 0.05). After seeding stage, ECs changed insignificantly (*P* > 0.05). At the 20–40 cm and 40–60 cm depths, ECs at seeding stage significantly differed from that of flowering stage (*P* < 0.05), and it was similar to the values of other stages (*P* > 0.05). Soil salinity at the 60–80 cm depth had no significant difference across various stages (*P* > 0.05). From seeding stage to flowering stage, soil salinity was effectively decreased at the depths of 0–20 cm, 20–40 cm and 40–60 cm, with the desalinization rates of 51%, 43% and 19%, respectively. After flowering stage, the phenomenon of salt gathering or salt returning occurred at the depths of 20–40 cm and 40–60 cm. At boll stage, only ECs at 0–20 cm decreased considerably compared to flowering stage (*P* < 0.05), and the desalinization rate of soil was 48%.

Soil salinity of the mildly and moderately salinized cotton field shows significant variation across different growth stages. The soil profile indicates the transition from surface accumulation to subsurface desalting. The depth of 60 cm from surface was a turnover point from significant desalinization to insignificant desalinization, even salt accumulation. Salinity of the surface soil in the moderately salinized plot was reduced to the level of mild salinization. For the mildly salinized plot, ECs of the surface soil decreased to the extent that salinization was alleviated. In relative term, the subsurface pipe drainage technique achieved a better improvement effect in moderately salinized plot.

### Variation in drainage water EC_w_ and pH value

**Figure 7:**
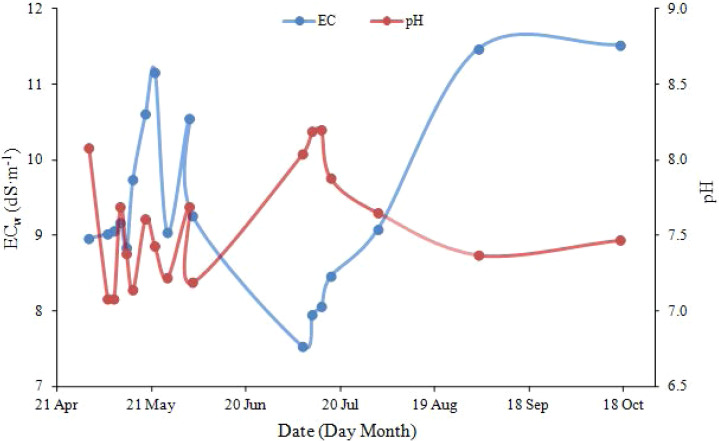
EC_w_ and pH value of drainage water from subsurface pipe.

EC_w_ and pH values of drainage water from subsurface pipe are presented in Fig. 7. At the initial stage of irrigation (May 1–22), the irrigation amount was small, and the concentration of salts leached by irrigation was high. As the irrigation amount increased (between May 22 and July 10), the concentration of the leached salts gradually decreased due to dilution from the large volume of irrigation water. The concentration of salts leached from the farmlands increased because of decreasing irrigation amount between July 10 and September 6 (Fig. 4). After the last irrigation on September 6, EC_w_ remained stable due to lack of irrigation water. EC_w_ of the drainage water varied between 7.53 and 11.66 dS m-1 (*i.e.,* −17%–27%). The pH value fluctuated within the range of 7.08–8.20, which was related to the irrigation amount, soil properties and drainage amount of subsurface pipes. The pH value in the whole drainage process was greater than 7, which indicates that water soluble salt ions (alkaline ions) were discharged and subsurface pipe drainage was conductive to the improvement of salinized soil. Soil salinity, soil texture, as well as the amount, frequency and quality of irrigation water, have certain influences on the desalinization effect. Further study needs to be undertaken for the determination of an optimal irrigation quota.

Subsurface pipe drainage can effectively reduce soil salinity and achieve the desalinization. Our findings are basically consistent with the previous studies (Christen & Skehan, 2001; Ritzema et al., 2008). The effect of the irrigation water was not explored, but would be expected to affect the desalinization of both the moderately and mildly salinized soils. The farmland irrigation water used in this study was from deep groundwater (depth: 300 m), which had a very low mineralization on average (EC: 0.35 dS m^−1^). Therefore, the irrigation water had a little effect on salinity. During the irrigation stage (between May 1 and September 6), the average precipitation was 0.457 mm d^−1^, while the average evapotranspiration was 3.659 mm d^−1^. The low precipitation could not compensate for the high evapotranspiration, so the effect of precipitation on salt leaching was negligible. The reduction of salinity of the farmlands was mainly by leaching in irrigation water. Based on the samples taken at seeding stage before irrigation, on bud stage, flowering stage and boll stage, the results of soil profile analysis shows that salts were effectively leached by irrigation, and the salinity of all soil layers was decreased. The most substantial decrease occurred in the soil layer of 0–20 cm, from 6.26 dS m^−1^ to 0.57 dS m^−1^. Whole salinity at the depths of 40–60 cm and 60–80 cm decreased in bud stage and flowering stage. The average desalinization rate of the mildly salinized soil was 22%, and that of the moderately salinized soil was 56%. These results indicated that soil desalinization was closely related to subsurface pipe drainage.

### Variation of cotton yield

**Figure 8:**
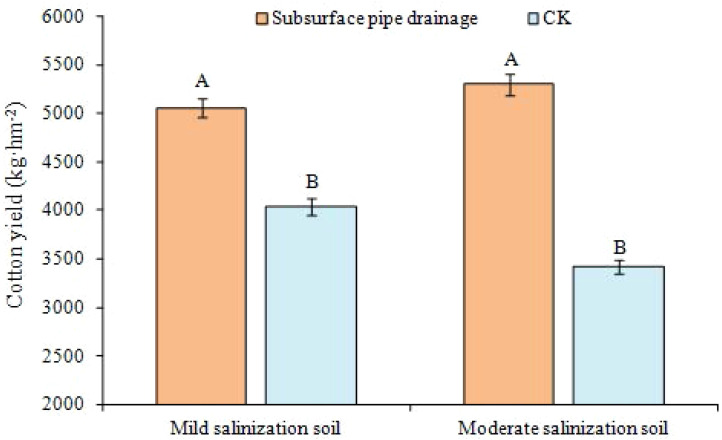
Cotton yield in different salinization soils. Data show the mean ± standard error (*n* = 3). Significant differences (*P* < 0.01) among treatments are indicated by different capital letters.

The yield comparison between the two experimental plots with different levels of salinization (Fig. 8) indicates that there was no significant difference in cotton yield (*P* > 0.01). However, the differences were extremely significant when they were compared with the CK plots (*P* < 0.01). The yield on mildly salinized soil was 5,059 kg ha^−1^, showing an increase of 25% compared with the corresponding CK plot. The yield increase was 55% in moderately salinized soil compared with the corresponding CK plot. Furthermore, stronger growth vigor was found in the treatments with subsurface pipe drainage than without subsurface pipe drainage, and the area of salinized farmlands decreased considerably. The emergence rate of cotton in the CK plots without subsurface pipe drainage was less than 20%, while that in the treatments with subsurface pipe drainage was 70% to 80% after first irrigation. Moreover, the death of seedlings after rain was reduced. These results indicate that crop yield increased significantly under the condition of subsurface pipe drainage.

### Engineering economy

The cost of draining the open ditch is **¥** 45,000 for five years of normal operation with three pipes of controlled width equivalent to an open ditch (requiring bottom silt removal once a year); the pipes only require a one-off investment of **¥** 42,000 in the first year. Meanwhile, the concealed pipe does not occupy surface arable land, while the open ditch covers 15 acres per kilometer. Based on a benefit of **¥**1,000 per acre, the piped drainage can save **¥** 75,000 over 5 years compared to open ditch drainage.

## Conclusions

The effect of shallow subsurface pipe drainage on salt level and crop yield was examined in an integrated analysis of the change of soil, drainage water and crop during crop growth stages under mulched drip irrigation. The study results have proved that salinized farmlands can be improved by subsurface pipe drainage. The following conclusions can be drawn: (1) Soil salinity was reduced in both mildly salinized land and moderately salinized land. The desalinization effect in a moderately salinized cotton field was greater than that in the mildly salinized cotton field. The highest desalinization rate of mildly and moderately salinized soils was 51% and 91%, respectively. (2) The desalinization in upper soil layers was more significant than in lower layers. The decrease of soil salinity at the depth of 0–20 cm was greater than that at other layers. The depth of 60 cm from surface was a turnover point from significant desalinization to insignificant desalinization, even salt accumulation. (3) The EC_w_ of subsurface pipe drainage water was greater than the EC of irrigation water, indicating that subsurface pipe drainage removes the salts from the salinized farmlands through irrigation. EC_w_ varied in the range of 7.53–11.66 dS m^−1^ and pH value varied from 7.08 to 8.20. These values varied with irrigation rate and other human induced factors, such as fertilizer application. (4) Compared with the two corresponding CK plots, crop yield increased significantly under the condition of subsurface pipe drainage. Crop yield in mild salinization increased about 25%. The crop yield in moderate salinization increased more than 50%.

Finally, the field experiment was limited by the labor-cost. Several years of research will be carried out to validate the desalinization effect of drip irrigation in combination with subsurface drainage. Several issues need to be further investigated to ensure adequate and detailed information on soil salinization improvement (Yang, Chen & Zhang, 2019). These include impacts of different irrigation volume and times, different soil texture, and different mineralization of irrigation water. Overall, this study quantifies the effects of shallow subsurface pipe drainage on improving soil salinization and crop yield. This engineering improvement has led to 330 ha soil salinization farmlands being restored to reduce soil salinity and increase crop yield. The approach provides an effective technical basis to policy making on water resource management and sustainable agricultural production of salinization lands in arid region.

## Supplemental Information

10.7717/peerj.12622/supp-1 Supplemental Information 1 Raw Data

## Additional Information and Declarations

### Data Availability

The following information was supplied regarding data availability: The raw measurements are available in the Supplementary File.
